# Impact of UPF2 on the levels of CD81 on extracellular vesicles

**DOI:** 10.3389/fcell.2024.1469080

**Published:** 2024-11-25

**Authors:** Chaehwan Oh, Krystyna Mazan-Mamczarz, Myriam Gorospe, Ji Heon Noh, Kyoung Mi Kim

**Affiliations:** ^1^ Department of Biological Sciences, Chungnam National University, Daejeon, Republic of Korea; ^2^ Laboratory of Genetics and Genomics, National Institute on Aging Intramural Research Program, National Institutes of Health, Baltimore, MD, United States; ^3^ Molecular Aging Biology Laboratory (MABL), Department of Biochemistry, College of Natural Science, Chungnam National University, Daejeon, Republic of Korea

**Keywords:** UPF2, CD81, extracellular vesicles, EV biogenesis, EV uptake, exosomes

## Abstract

Extracellular vesicles (EVs) are involved in cell-to-cell communication. Following uptake, EV cargo molecules, including DNA, RNA, lipids, and proteins, influence gene expression and molecular signaling in recipient cells. Although various studies have identified disease-specific EV molecules, further research into their biogenesis and secretion mechanisms is needed for clinical application. Here, we investigated the role of UPF2 in regulating the biogenesis and components of EVs. Notably, UPF2 promoted the expression of CD81, a membrane protein marker of EVs, as UPF2 silencing decreased CD81 levels in EVs, both inside the cell and secreted. In contrast, the expression levels of CD63 increased, without altering the size or numbers of EVs. In addition, reducing UPF2 levels did not affect the total number of EVs but lowered production of CD81-positive EVs and reduced the efficiency of uptake by recipient cells. Collectively, our findings uncover a novel function for UPF2 in regulating the production of CD81 and changing EV properties.

## Introduction

Most cells secrete EVs that contain a variety of molecules, including proteins, DNA, RNA, and bioactive lipids. Secreted EVs can be taken up by recipient cells, where they can alter signaling cascades and modify gene expression programs (Chen et al., 2018; Kim et al., 2017; Ratajczak et al., 2006). EVs are classified in different ways according to size and genesis; traditionally, they have been divided into exosomes, microvesicles, and apoptotic bodies (Borges et al., 2013; Kim et al., 2017). The formation and secretion of exosomes require the cooperation of several molecules, including Rab proteins, the endosomal sorting complex required for transport (ESCRT), tetraspanins, and sphingomyelinases (Chairoungdua et al., 2010; Gerber et al., 2015; Gould et al., 2003; Henne et al., 2011; Hsu et al., 2010; Ostrowski et al., 2010; Perez-Hernandez et al., 2013; Radulovic and Stenmark, 2018; Savina et al., 2002; Van Niel et al., 2011; Verweij et al., 2011). In this study, we mainly examined exosomes derived from multivesicular bodies (MVBs). CD81, CD63, and CD9 proteins primarily accumulate in EVs and are used as representative EV markers (Escola et al., 1998; Thery et al., 1999). However, some studies suggest that isolated EVs are quite heterogeneous, with different levels of surface proteins that alter EV properties (Kowal et al., 2016; Meng et al., 2023). For example, CD9 and CD63 proteins are known to have different plasma membrane localization and internalization kinetics (Fan et al., 2023; Mathieu et al., 2021).

EVs can be detected in various body fluids, including blood, urine, breast milk, lymph, and saliva (Dai et al., 2020; Xu et al., 2016; Zlotogorski-Hurvitz et al., 2015). Moreover, EVs containing disease-specific molecules have been obtained from patients with diseases such as cancer, hypertension, and cardiovascular pathologies (Zhou et al., 2020). Because EVs can be easily collected from the blood of patients, they are being actively studied for their value in the diagnosis and prognosis of diseases. Indeed, reports of disease diagnosis using EVs have been increasing over the past decade (Zhou et al., 2020). For example, Melo et al. (2015) found increased glypican-1 (GPC1) levels in EVs isolated from the blood of patients with early-stage pancreatic cancer, and proposed that circulating GPC1^+^ exosome levels could predict the stage of pancreatic cancer (Melo et al., 2015). EVs have a bilayer structure that allows them to safely transport their cargo over long distances. Therefore, scientists are attempting to exploit them as drug delivery vehicles. Yim et al. (2016) reported the successful loading of target proteins into EVs using an optically reversible protein–protein interaction module based on plant proteins that respond to blue light (Yim et al., 2016). This and other studies underscore the notion that understanding the properties of EVs is necessary to exploit their full potential as therapeutic vehicles. Unfortunately, the mechanisms of EV biogenesis, secretion, organ-specific targeting, and uptake are poorly understood.

Recently, Notaras et al. (2020) found that knockdown of UPF2 in neurons decreased the levels of Glutamate Receptor 1 levels and also reduced its surface localization. This discovery raised the possibility that UPF2 may affect the level or localization of plasma membrane proteins (Notaras et al., 2020). Unlike CD63, CD81 and CD9 are tetraspanin proteins that mostly translocate to the plasma membrane and are heavily included in EVs formed by budding (Mathieu et al., 2021). UPF2 plays an important role in nonsense-mediated mRNA decay (NMD), a mechanism that removes abnormal mRNA containing premature termination codons (PTCs). NMD is one of the mechanisms by which the quality of mRNA is tightly controlled within a cell to prevent the production of defective or potentially toxic proteins (Arraiano and Maquat, 2003; Behm-Ansmant et al., 2007; Doma and Parker, 2007). UPF2 is one of the protein constituents of the exon junction complex that binds upstream of the exon–exon junctions after RNA splicing; it binds UPF1 and plays an important role in recognizing mRNAs containing PTCs (Arraiano and Maquat, 2003; Behm-Ansmant et al., 2007; Cheng and Maquat, 1993; Doma and Parker, 2007; He and Jacobson, 2015; Maquat, 2004). Here, we set out to investigate the link between UPF2 and EV surface proteins and find that silencing UPF2 reduces CD81 expression. Interestingly, EVs derived from UPF2-silenced HeLa cells were less efficiently taken up by recipient cells due to the absence of CD81. We propose that UPF2 modulates EV surface markers and EV function.

## Materials and methods

### Cell culture and transfection

WI-38 human diploid fibroblasts (HDFs) were obtained from Coriell Cell Repositories (NJ, United States), and human cervical carcinoma HeLa cells were from ATCC (VA, United States). HeLa and WI-38 cells were cultured in Dulbecco’s modified Eagle’s medium (Welgene, Gyeongsan, South Korea) supplemented with 10% fetal bovine serum (Gibco, MA, United States) and 1% antibiotic-antimycotic solution (Anti-Anti, Gibco). WI-38 cells were additionally cultured in the presence of 1% non-essential amino acids (Gibco; BD Biosciences) and used with a population doubling level (PDL) of 25. For specific silencing experiments, HeLa cells were transfected with 50nM of small interfering RNA (siRNA) using Lipofectamine 2000 (Invitrogen, CA, United States). After 72 h of incubation, cells were harvested and assays were performed. The gene-specific siRNA sequences are shown in Table 1. For human CD81 overexpression, HeLa cells were transfected with pCMV6-Entry or pCMV6-Entry-Myc-DDK-CD81 plasmid RC217508 (OriGene Technologies, Inc., MD, United States) using Lipofectamine 2000. After 48 h, cells were harvested and assays were performed.

**TABLE 1:** Sequence of specific siRNAs.

Target Gene	siRNA sequence (5’ to 3’)
*Control*	UUC​UCC​GAA​CGU​GUC​ACG​U
*UPF2 #1*	GCA​ACG​AAG​UGG​UGA​AUC​U
*UPF2 #2*	CAA​GAA​GUG​GAU​GAG​AAU​A
*UPF1*	GAU​GCA​GUU​CCG​CUC​CAU​U
*CD81*	CAC​GUC​GCC​UUC​AAC​UGU​A

### Cell lysates preparation and Western blot analysis

Whole-cell lysates were prepared using M-PER™ Mammalian Protein Extraction Reagent (Thermo Fisher Scientific, MA, United States) supplemented with Halt™ Protease and Phosphatase Inhibitor Cocktail (Thermo Fisher Scientific). Subsequently, NuPAGE™ LDS Sample Buffer (Thermo Fisher Scientific) supplemented with 5% 2-Mercaptoethanol (Sigma-Aldrich, MO, United States) was added; 2-Mercaptoethanol was not added to the samples used to detect CD63 and CD81 proteins by Western blotting. The same amount of lysates were electrophoresed in NuPAGE™ 4%–12%, Bis-Tris Mini protein Gel (Invitrogen) or Novex™ WedgeWell™ 6%, Tris-Glycine Mini Protein Gel (Invitrogen). Subsequently, the gels were transferred to a nitrocellulose membrane using iBlot™ 2 Transfer Stacks (Invitrogen). For EV samples, the membranes with transferred proteins were then stained with Ponceau S (Sigma-Aldrich) to monitor the evenness in loading and transfer of samples. After blocking with 5% skim milk, membranes were incubated with primary antibodies recognizing UPF2 (Abcam, United Kingdom), CD81 (Santa Cruz Biotechnology), CD63 (Santa Cruz Biotechnology), HSP90 (Santa Cruz Biotechnology), GAPDH (Santa Cruz Biotechnology), ACTB (Santa Cruz Biotechnology), FLAG (Sigma-Aldrich), or UPF1 (Cell Signaling Technology). Following incubation with the respective secondary antibodies (Sigma-Aldrich), protein signals were visualized using enhanced chemiluminescence (Thermo Fisher Scientific) and a KwikQuant Imager (Kindle Biosciences, LLC).

### Isolation of total EVs, measurement of EV size and concentration

For EV isolation, HeLa cells were cultured in exosome-depleted medium for 24 h before harvesting: Dulbecco’s modified Eagle’s medium (Welgene) supplemented with 10% exosome-depleted fetal bovine serum (Gibco) and 1% antibiotic-antimycotic solution (Gibco). Total EV samples were then isolated from conditioned medium using ExoQuick-TC (System Biosciences, CA, United States) according to the manufacturer’s instructions. Briefly, conditioned medium was centrifuged at 3,000 × *g* for 15 min to remove cell debris, after which it was filtered using a Millex-GV Syringe Filter Unit (0.22 μm, Millipore, MA, United States) and concentrated using an Amicon^®^ Ultra-15 Centrifugal Filter Unit (10 KDa cutoff, Millipore). Thereafter, an appropriate volume of ExoQuick-TC was added to the samples, which were then incubated at 4°C for ≥12 h and centrifuged at 3,400 × *g* for 20 min at 4°C. Next, Dulbecco’s phosphate-buffered saline (DPBS; filtered using a 0.22 μm filter) was added to the EV pellet. NuPAGE™ LDS Sample Buffer was then added for western blotting. For size and concentration analysis of EVs, isolated EVs were diluted with a total volume of 1 mL DPBS (filtered using a 0.22-μm filter) and analyzed using a NanoSight NS300 instrument (Malvern Panalytical, United Kingdom) or ZetaView PMX 430 (Particle Metrix, Germany).

### EV staining with PKH26 dye

For experiments on EV uptake by recipient cells, same number of EVs in each sample were stained using PKH26GL Red Fluorescent Cell Linker Kits for General Cell Membrane labeling (Sigma-Aldrich) according to the manufacturer’s instructions. EVs were incubated with Diluent C and PKH26 dye for 5 min in a dark chamber. Next, 1% bovine serum albumin was added to stop the staining reaction. The samples were then loaded onto an Amicon^®^ Ultra-15 Centrifugal Filter Unit (10 KDa cutoff, Millipore) and centrifuged. The unlabeled dye that passed through the filter was removed, and only the EVs retained on the filter were used. Next, the labeled EVs were diluted with exosome-depleted medium. After adding the labeled EVs to the cells, they were incubated at 4°C in the dark for 30 min, washed with DPBS, incubated with exosome-depleted medium for 1 h at 37°C in a CO_2_ incubator, and fixed with 4% paraformaldehyde. Nuclei were stained with ProLong^®^ Gold Antifade reagent and DAPI (Invitrogen). Images were acquired using an EVOS M5000 Imaging System (Invitrogen), LSM 880 with Airyscan (Carl Zeiss, Germany), or K1-Fluo (Nanoscope Systems, South Korea). The mean fluorescence intensity (MFI) for the PKH26 dye signal was calculated using ImageJ.

### RNA isolation, reverse transcription, and quantitative real time PCR

Total RNA was extracted using the TRI™ Reagent solution (Invitrogen). After ethanol precipitation, the samples were treated with DNase I (Thermo Fisher Scientific) at 37°C for 30 min to remove DNA. cDNA was synthesized using Random Hexamer Primer (Thermo Fisher Scientific) and RevertAid Reverse Transcriptase (Thermo Fisher Scientific). RT-qPCR analysis was performed using gene-specific primers, KAPA SYBR^®^ FAST (Kapa Biosystems, United Kingdom), and QuantStudio Real-Time PCR instrument (Applied Biosystems, United States). The sequences of the specific primers used for qPCR are in .

### Statistical analysis

Experimental results were expressed as means ± SEM. The Student's two-tailed *t*-test and one-way analysis of variance with Tukey’s *post hoc* test were performed using GraphPad Prism 8 software (GraphPad, San Diego, United States).

## Results

### UPF2 silencing decreases CD81 and increases CD63 in EVs

Recent reports have shown that UPF2 regulates the levels and localization of plasma membrane proteins (Notaras et al., 2020). We thus hypothesized that the expression of CD81, a surface protein and marker of EVs, might be regulated by UPF2. To investigate this possibility, we reduced UPF1 and UPF2 levels in HeLa cells using specific siRNAs. While silencing UPF1 did not alter CD81 expression levels, we unexpectedly found that silencing UPF2 lowered the levels of CD81 protein in HeLa cell (Supplementary Figure S1A, *left*). Using siRNAs targeting different regions of UPF2 (UPF2 #2 siRNA), we confirmed the decrease in CD81 protein levels (Supplementary Figure S1A, *right*). This finding suggested that UPF2 regulates the levels of CD81 by mechanisms independent of the NMD pathway.

**FIGURE 1:**
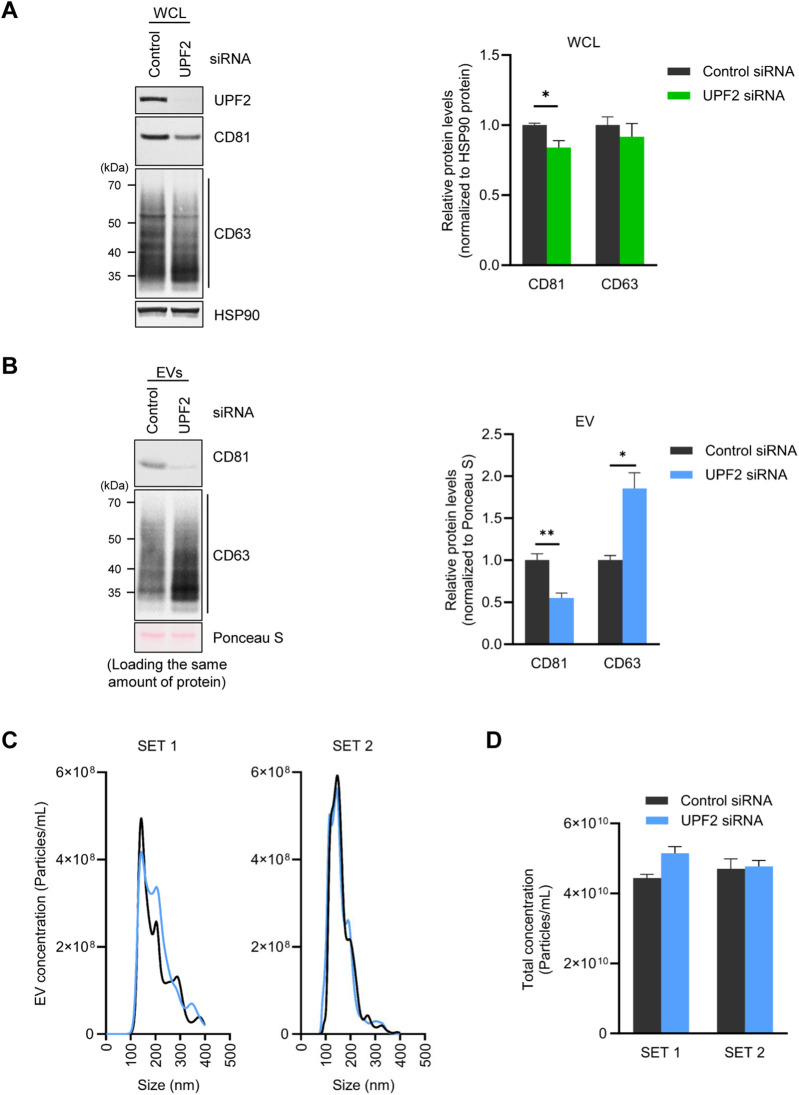
UPF2 silencing reduces CD81 protein in cells and extracellular vesicles (EVs). **(A–D)** HeLa cells were transfected with control or UPF2 siRNA. Afterwards, cells and EVs were harvested 72 h later. **(A)** CD81 expression levels were assessed in whole-cell lysates (WCL) by western blotting using an antibody recognizing CD81 (Left). CD81 and CD63 signals were quantified and normalized to HSP90 signals (Right). **(B)** EVs were isolated from HeLa cells transfected with control or UPF2 siRNA using ExoQuick-TC. The same amounts of EV proteins were loaded, and CD81 and CD63 expression levels were assessed in EVs by western blotting (Left). The quantification of CD81 and CD63 signals was normalized to the Ponceau S signal (Right). **(C)** The size distribution of isolated EVs was assessed using a Nanosight instrument (Malvern Panalytical). **(D)** Total EV concentration was plotted as a bar graph; HSP90, loading control. Data in **(A, B)** represent the mean ± S.E.M. of three independent experiments. *P ≤ 0.05, **P ≤ 0.01.

We focused on investigating the role of UPF2 in regulating CD81 expression on EV membranes. First, using western blot analysis, we investigated whether UPF2 knockdown affected the levels of CD81 and CD63, another EV membrane marker. As shown, silencing UPF2 markedly reduced the levels of intracellular CD81 protein, while the levels of CD63 remained unchanged (Figure 1A). To further assess the impact of UPF2 silencing, we prepared cytosolic and membrane proteins to examine the expression levels of CD81, a cell surface-localized tetraspanin protein. Notably, both cytosolic and membrane fractions showed decreased CD81 levels after UPF2 silencing (Supplementary Figure S1B). To investigate whether the decrease in CD81 protein by UPF2 silencing was the result of a decrease in *CD81* mRNA, we used RT-qPCR analysis to assess the steady-state levels of *CD81* mRNA; as shown, the levels of *CD81* mRNA remained unchanged after UPF2 silencing (Supplementary Figure S1C). We then investigated whether changing intracellular CD81 levels by UPF2 influenced EV biogenesis and secretion; as shown, silencing UPF2 decreased CD81 protein levels on EVs while the levels of CD63 increased (Figure 1B). Notably, nanoparticle tracking analysis revealed no substantial change in the total number of EVs released by the different silencing groups (Figures 1C, D).

EVs secreted by cells are highly heterogeneous, with each EV containing different levels of CD81 and CD63 (Kowal et al., 2016; Mathieu et al., 2021). To investigate if there were different numbers of EVs containing CD81 or CD63 in the general cell population, we separated total EVs into CD81-positive and CD81-negative EVs by magnetic separation. The results show that CD81-positive EVs contained both CD81 and CD63 proteins, whereas CD81-negative EVs contained CD63 only (Supplementary Figure S2A). We then determined whether UPF2 silencing alters the population of secreted EVs. As anticipated, UPF2 knockdown increased the population of CD81-negative EVs and decreased the CD81-positive EV population (Supplementary Figure S2B). Taken together, these findings show that silencing UPF2 reduces CD81 protein levels in cells and EVs without affecting the overall number and size of secreted EVs, suggesting that CD81-negative EVs increase proportionately.

### UPF2 silencing lowers CD81 production, CD81-containing EVs, and EV uptake efficiency

**FIGURE 2:**
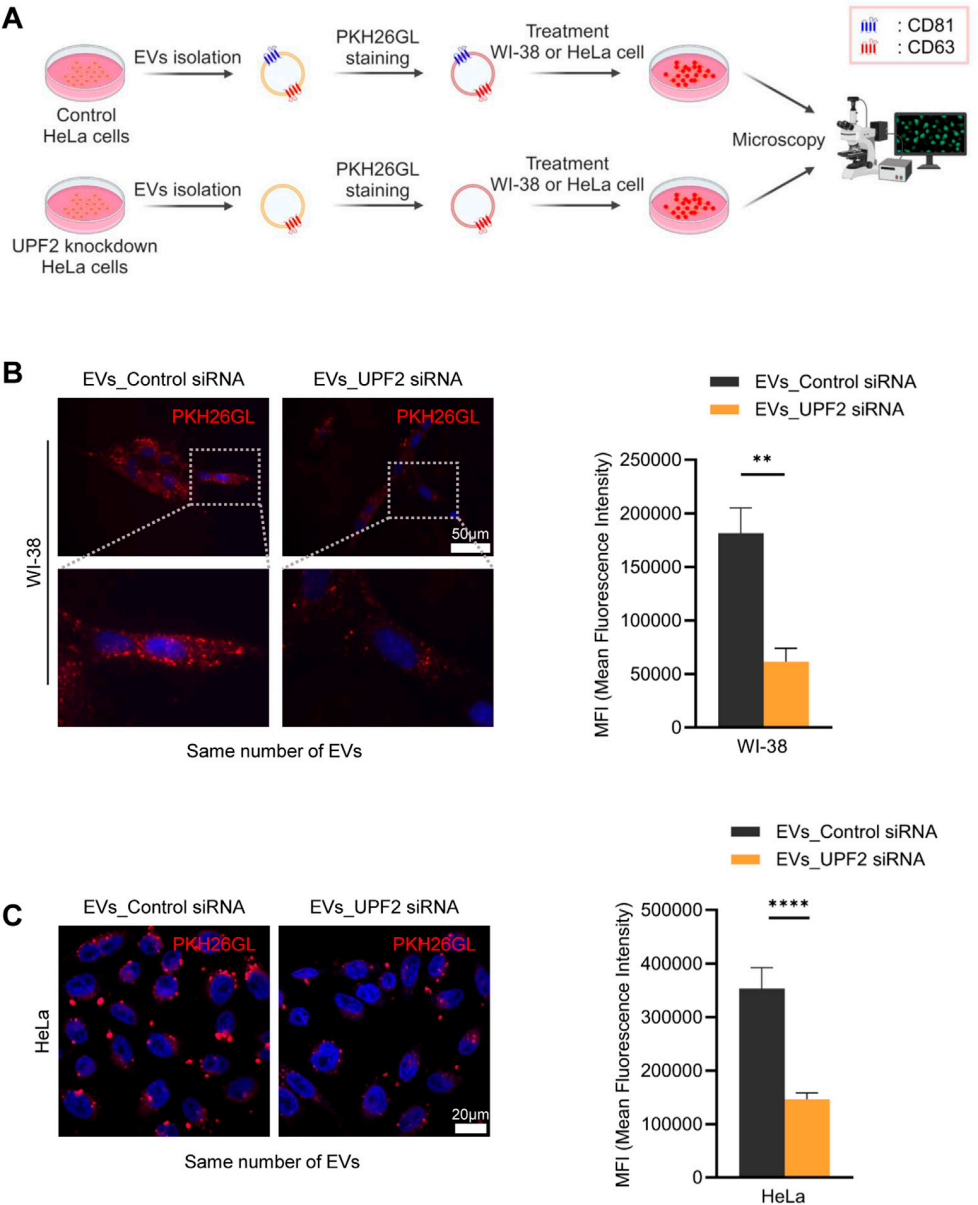
Extracellular vesicles (EVs) secreted from UPF2 silenced HeLa cells have different characteristics. **(A)** Schematic illustrating the investigation of the EV uptake efficiency of recipient cells. EVs were isolated from HeLa cells transfected with control (EVs_Control siRNA) or UPF2 siRNA (EVs_UPF2 siRNA). Then, the same number of EVs were stained using PKH26 dye. The stained EVs were incubated with WI-38 or HeLa cells and examined for uptake into recipient cells by fluorescence microscopy. **(B)** Fluorescence microscopy images of WI-38 cells. Red signals indicate EVs taken up by WI-38 cells, and blue signals represent DAPI. Below are enlarged images of the fields indicated by the white squares (Left). Quantification of red fluorescence intensity normalized to the number of nuclei (Right). Scale bar = 50 μm. **(C)** Images showing the uptake of PKH26-stained EVs into HeLa cells (Left). These images were taken using a confocal microscope (Carl Zeiss). Scale bar = 20 μm. Quantification of red fluorescence signals normalized to the number of nuclei (Right). Mean fluorescence intensity (MFI) was calculated using Image J. **P ≤ 0.01, ****P ≤ 0.0001.

Silencing UPF2 reduces the levels of CD81, a membrane protein constituent of EVs. On this basis, we hypothesized that the decline in CD81 levels on EVs membrane could affect the characteristics of EVs, particularly the efficiency of EV uptake by recipient cells. We first isolated EVs from HeLa cells in control and UPF2-silenced populations, and labeled the isolated EVs with PKH26, a red fluorescent linker. The fluorescently labeled EVs were then incubated with HeLa cells or human WI-38 lung fibroblasts for 30 min to determine their uptake by recipient cells (Figure 2A). Notably, incubation with EVs derived from HeLa cells in which UPF2 was silenced (EVs_UPF2 siRNA) caused significantly reduced fluorescence (reduced uptake) in WI-38 (Figure 2B) and HeLa cells (Figure 2C) compared with incubations with those EVs derived from control (EVs_Control siRNA), which is due to reduced EV uptake by recipient cells. These results indicate that EVs secreted from UPF2-silenced cells impair overall uptake of EVs by recipient cells.

**FIGURE 3:**
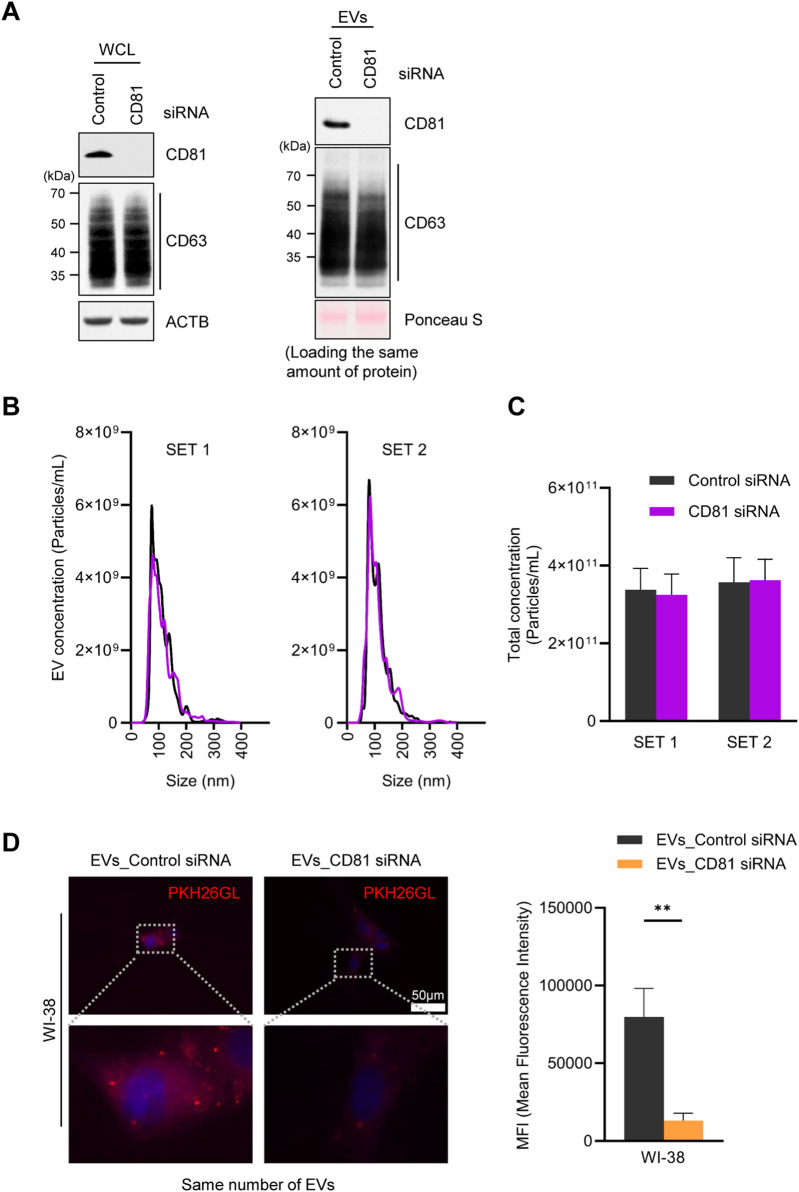
Absence of CD81 in extracellular vesicles (EVs) decreases uptake efficiency. **(A)** Protein expression levels in CD81 silenced cells and EVs were analyzed by western blotting by loading equal amount of protein. **(B)** Size distribution of EVs derived from control and CD81 silenced cells was assessed using nanoparticle tracking analysis (NTA). **(C)** Concentration of total EVs secreted from control or CD81 siRNA-treated cells. **(D)** The same number of EVs were stained using PKH26 dye and incubated with WI-38 cells. The red signal of EVs taken up by WI-38 cells was measured by fluorescence microscopy (Left). Scale bar = 50 μm. Red intensity was normalized to the number of nuclei (Right). Mean fluorescence intensity (MFI) was calculated using Image J. The experiments in **(A)** were performed in duplicate. **p ≤ 0.01.

In light of earlier evidence that co-culture of cells, EVs, and CD81 antibodies reduced EV uptake by bone marrow dendritic cells (Morelli et al., 2004), we hypothesized that the presence of CD81 on EVs may be important for their delivery to recipient cells. To test this hypothesis, we silenced CD81 and measured EV uptake efficiency. Western blot analysis revealed that CD81 knockdown reduced CD81 expression in both cells and EVs, without affecting CD63 expression levels (Figure 3A). Interestingly, silencing CD81 in HeLa cells did not significantly affect EV particle number or size (Figures 3B, C). To investigate the uptake capacity of EVs derived from HeLa cells in which CD81 was silenced, we co-cultured the isolated EVs with WI-38 cells. As observed, EVs from CD81-silenced cells (EVs_CD81 siRNA) showed lower uptake efficiency compared with that of control EVs (EVs_Control siRNA) (Figure 3D). These results suggest that the presence of CD81 on EVs is important for uptake by recipient cells, and that CD81-negative EVs have lower uptake efficiency than CD81-positive EVs.

### CD81 overexpression contributes to an increase in the number of EVs and their uptake capacity

**FIGURE 4:**
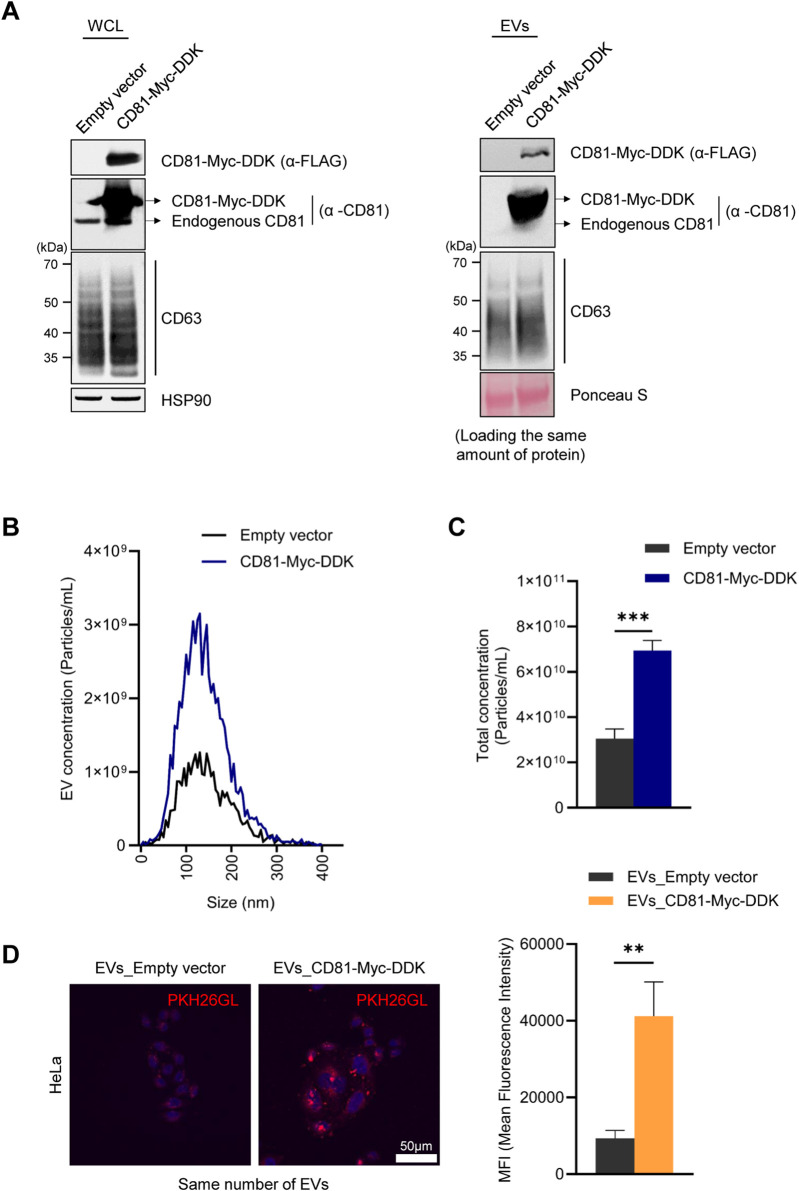
CD81-overexpressing extracellular vesicles (EVs) have increased uptake by recipient cells. **(A)** FLAG and CD81 expression levels in the CD81-overexpressing cells and EVs were analyzed by western blotting. The same amount of protein was loaded for western blotting. **(B)** The size distribution of EVs derived from CD81-overexpressing cells was assessed using a ZetaView instrument (Particle Metrix). **(C)** Concentration of total EVs secreted from control or CD81-overexpressing cells. **(D)** The same number of EVs were stained using PKH26 dye. The uptake efficiency of EVs into HeLa cells was evaluated using confocal microscopy (Nanoscope Systems) (Left). Scale bar = 50 μm. Red fluorescence intensity was normalized to the number of nuclei (Right). Mean fluorescence intensity (MFI) was calculated using Image J. The experiments in **(A, B)** were performed in triplicate. Data in **(C)** represent the mean ± S.E.M. of three independent experiments. **P ≤ 0.01, ***P ≤ 0.001.

To gain further information regarding the importance of CD81 in EV uptake capacity, we overexpressed CD81 in HeLa cells to generate CD81-overexpressing EVs. As observed after silencing CD81, overexpressing CD81 did not induce significant changes in CD63 levels in HeLa cells or HeLa-derived EVs (Figure 4A). However, unexpectedly, CD81-overexpressing cells released more than twice as many EVs than control cells (Figures 4B, C), suggesting that CD81 may contribute to generating and releasing EVs. To investigate whether CD81 overexpression enhances EV uptake capacity, we treated HeLa cells with EVs obtained from control or CD81-overexpressing cells. As shown, EVs from CD81-overexpressing cells were more efficiently taken up by recipient cells (Figure 4D). Collectively, these data suggest that CD81, which is present on the EV membrane, is an essential protein for uptake by recipient cells. Taken together, our study indicates that UPF2 can promote uptake efficiency at least in part by elevating CD81 levels.

## Discussion

In recent years, EVs have been investigated as diagnostic and therapeutic agents, and their promising role in clinical applications has been highlighted (Zhou et al., 2020). In addition, efforts are ongoing to increase EV productivity and improve their delivery efficiency through engineering approaches to maximize their utilization (Boussadia et al., 2018; Fukuta et al., 2020; Nakase and Futaki, 2015; Rocha et al., 2019). However, the molecular mechanisms underlying the production and secretion of EVs with different characteristics are still poorly understood. A comprehensive understanding of the physiological biology of EVs is crucial for successfully developing and applying therapeutics utilizing EVs. Specifically, in the present study, we identified the novel functions of UPF2 in regulating EV production and secretion.

Analysis of the potential relationship between UPF2 and EV function, revealed that silencing UPF2 (but not silencing UPF1) significantly reduced the levels of the major EV protein CD81 (Supplementary Figure S1). This finding was unexpected, given that silencing UPF2 increases expression of mRNAs regulated by NMD, suggesting that the rise in CD81 production was not directly related to NMD-related activities of UPF2. Silencing UPF2 led to the secretion of many CD81-negative EVs (Figure 1B; Supplementary Figure S2 **)**, while the levels of CD63 in cells did not significantly change (Figure 1A). These findings support the classification of EVs into subtypes based on specific markers such as CD81 and CD63 (Kowal et al., 2016) and agree with previous studies (Mathieu et al., 2021) showing that EVs carrying tetraspanins, such as CD9, CD81, and small amounts of CD63 protein, primarily originated from the plasma membrane, whereas those bearing CD63 and small amounts of CD9 were mainly derived from intraluminal vesicles. Furthermore, CD81^−^ or CD9-positive EVs were found to be smaller than CD63-positive EVs (Mathieu et al., 2021).

Given that most CD81-positive EVs are formed through membrane budding, we hypothesize that UPF2 prevents the internalization of CD81 at the plasma membrane and the generation of CD81-positive EVs. This possibility supports a novel function of UPF2 that does not involve NMD, since UPF2 silencing reduces CD81 abundance in the cell. Given that CD81 protein is known to be a specific EV marker released through an ESCRT-independent pathway, while CD63 protein is considered a common EV marker regardless of the ESCRT complex (Xu et al., 2016), UPF2 appears implicated in maintaining the balance of specific EV subtypes. Furthermore, the absence of differences in the number of EVs released in conditions of reduced UPF2 levels strongly supports the hypothesis that UPF2 functions as a qualitative switch rather than a quantitative regulator. Further studies are necessary to test this hypothesis.

**FIGURE 5:**
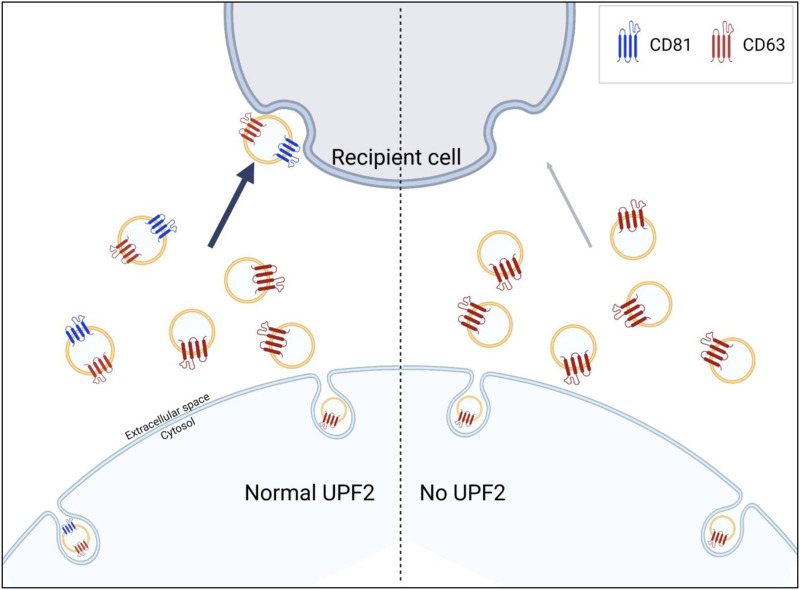
UPF2 contributes to the formation of extracellular vesicles (EVs) more efficiently taken up by recipient cells. UPF2 regulates the secretion of specific EV subgroups from cells. Under UPF2-deficient conditions, the secretion of EV subgroups is altered with a decrease in the release of CD81-positive EVs and a compensatory increase in the release of CD81-negative EVs. As a result, the reduction in the release of CD81-positive EVs, which exhibit higher uptake efficiency, decreases overall uptake efficiency.

The pathways by which EVs are internalized into recipient cells are highly cell type-dependent, making it difficult to conclude that EV uptake occurs exclusively through target-specific (e.g., receptor-dependent endocytosis) or non-specific pathways (e.g., pinocytosis) (Bonsergent et al., 2021; Costa Verdera et al., 2017; Mulcahy et al., 2014; Svensson et al., 2013). However, in the present study, we found that CD81-positive EVs elicit higher uptake. Additionally, the reduction of UPF2 in cells decreased the release of CD81-positive EVs, resulting in a decrease in uptake efficiency despite the secretion of a similar number of EVs with a comparable size distribution (Figures 2, 3). Similarly, EVs containing more CD81 had higher uptake efficiency despite the same number of particles (Figure 4), suggesting that, at least in HeLa cells, the released EVs are predominantly taken up by recipient cells through a target-specific rather than a non-specific pathway (Figure 5). CD81 on EVs may act as a ligand or receptor to initiate the uptake pathway; nevertheless, evidence supporting this hypothesis is still limited and requires further study.

In closing, we report that UPF2 influences EV biology. Although the mechanisms where by UPF2 promotes CD81 expression and EV function were not elucidated in the current study, we hypothesize that molecules interacting with UPF2, such as other RNAs or RNA-binding proteins, may indirectly regulate the production of CD81, and hence its inclusion in cell membrane and subsequently in EVs. Furthermore, our study supports the notion that the specific composition of EVs is regulated individually. Gaining further insight into the biogenesis and secretion of EVs will set the stage for further understanding the biology of EVs and leveraging its diagnostic, prognostic, and therapeutic potential.

## Data Availability

The raw data supporting the conclusions of this article will be made available by the authors, without undue reservation.

## Supplementary material

SUPPLEMENTARY FIGURE S1 Effect of NMD factor deficiency on CD81 expression in HeLa cells. **(A)** HeLa cells were transfected with control, UPF2 #1 (targeting the open reading frame region of UPF2, *left*), UPF2 #2 (targeting the 3’ untranslated region of UPF2, *right*) or UPF1 siRNAs, and EVs markers were assessed using Western blot analysis. **(B)** The expression levels of CD81 were assessed in cytosolic and membrane lysates using Western blot analysis. **(C)** Steady-state levels of *CD81* mRNA were quantified by RT-qPCR analysis and normalized to *ACTB* mRNA levels; UPF2 and UPF1, NMD factors; CD81 and CD63, EVs markers; GAPDH, a loading control or cytosolic marker; *SC35 1.6 kb* and *GADD45A* mRNA, NMD substrates; *GAPDH* mRNA, a negative control. Data in **(C)** represent the mean ± S.E.M. of three independent experiments. **P* ≤ 0.05.

SUPPLEMENTARY FIGURE S2 Knockdown of UPF2 decreases the secretion of CD81-positive EVs and increases the secretion of CD81-negative EVs. **(A)** Total EVs were separated into CD81-positive and -negative EVs using MACS, and the expression levels of CD81 and CD63 were analyzed by Western blot analysis. **(B)** Total EVs derived from Control or UPF2 knockdown HeLa cells were separated as CD81-positive and -negative EVs using MACS. The separated EVs were loaded with same amounts of protein and the expression levels of CD81 and CD63 in separated EVs were determined by Western blot analysis.

## References

[B1] Arraiano C. M. Maquat L. E. (2003). Post-transcriptional control of gene expression: effectors of mRNA decay. Mol. Microbiol. 49 (1), 267–276. 10.1046/j.1365-2958.2003.03545.x 12823827

[B2] Behm-Ansmant I. Kashima I. Rehwinkel J. Sauliere J. Wittkopp N. Izaurralde E. (2007). mRNA quality control: an ancient machinery recognizes and degrades mRNAs with nonsense codons. FEBS Lett. 581 (15), 2845–2853. 10.1016/j.febslet.2007.05.027 17531985

[B3] Bonsergent E. Grisard E. Buchrieser J. Schwartz O. Thery C. Lavieu G. (2021). Quantitative characterization of extracellular vesicle uptake and content delivery within mammalian cells. Nat. Commun. 12 (1), 1864. 10.1038/s41467-021-22126-y 33767144 PMC7994380

[B4] Borges F. T. Reis L. A. Schor N. (2013). Extracellular vesicles: structure, function, and potential clinical uses in renal diseases. Braz J. Med. Biol. Res. 46 (10), 824–830. 10.1590/1414-431X20132964 24141609 PMC3854311

[B5] Boussadia Z. Lamberti J. Mattei F. Pizzi E. Puglisi R. Zanetti C. (2018). Acidic microenvironment plays a key role in human melanoma progression through a sustained exosome mediated transfer of clinically relevant metastatic molecules. J. Exp. Clin. Cancer Res. 37 (1), 245. 10.1186/s13046-018-0915-z 30290833 PMC6173926

[B6] Chairoungdua A. Smith D. L. Pochard P. Hull M. Caplan M. J. (2010). Exosome release of β-catenin: a novel mechanism that antagonizes Wnt signaling. J. Cell. Biol. 190 (6), 1079–1091. 10.1083/jcb.201002049 20837771 PMC3101591

[B7] Chen G. Huang A. C. Zhang W. Zhang G. Wu M. Xu W. (2018). Exosomal PD-L1 contributes to immunosuppression and is associated with anti-PD-1 response. Nature 560 (7718), 382–386. 10.1038/s41586-018-0392-8 30089911 PMC6095740

[B8] Cheng J. Maquat L. E. (1993). Nonsense codons can reduce the abundance of nuclear mRNA without affecting the abundance of pre-mRNA or the half-life of cytoplasmic mRNA. Mol. Cell. Biol. 13 (3), 1892–1902. 10.1128/mcb.13.3.1892 8441420 PMC359503

[B9] Costa Verdera H. Gitz-Francois J. J. Schiffelers R. M. Vader P. (2017). Cellular uptake of extracellular vesicles is mediated by clathrin-independent endocytosis and macropinocytosis. J. Control Release 266, 100–108. 10.1016/j.jconrel.2017.09.019 28919558

[B10] Dai J. Su Y. Zhong S. Cong L. Liu B. Yang J. (2020). Exosomes: key players in cancer and potential therapeutic strategy. Signal Transduct. Target Ther. 5 (1), 145. 10.1038/s41392-020-00261-0 32759948 PMC7406508

[B11] Doma M. K. Parker R. (2007). RNA quality control in eukaryotes. Cell. 131 (4), 660–668. 10.1016/j.cell.2007.10.041 18022361

[B12] Escola J. M. Kleijmeer M. J. Stoorvogel W. Griffith J. M. Yoshie O. Geuze H. J. (1998). Selective enrichment of tetraspan proteins on the internal vesicles of multivesicular endosomes and on exosomes secreted by human B-lymphocytes. J. Biol. Chem. 273 (32), 20121–20127. 10.1074/jbc.273.32.20121 9685355

[B13] Fan Y. Pionneau C. Cocozza F. Boelle P. Y. Chardonnet S. Charrin S. (2023). Differential proteomics argues against a general role for CD9, CD81 or CD63 in the sorting of proteins into extracellular vesicles. J. Extracell. Vesicles 12 (8), e12352. 10.1002/jev2.12352 37525398 PMC10390663

[B14] Fukuta T. Nishikawa A. Kogure K. (2020). Low level electricity increases the secretion of extracellular vesicles from cultured cells. Biochem. Biophys. Rep. 21, 100713. 10.1016/j.bbrep.2019.100713 31828227 PMC6889636

[B15] Gerber P. P. Cabrini M. Jancic C. Paoletti L. Banchio C. von Bilderling C. (2015). Rab27a controls HIV-1 assembly by regulating plasma membrane levels of phosphatidylinositol 4,5-bisphosphate. J. Cell. Biol. 209 (3), 435–452. 10.1083/jcb.201409082 25940347 PMC4427790

[B16] Gould S. J. Booth A. M. Hildreth J. E. (2003). The Trojan exosome hypothesis. Proc. Natl. Acad. Sci. U. S. A. 100 (19), 10592–10597. 10.1073/pnas.1831413100 12947040 PMC196848

[B17] He F. Jacobson A. (2015). Nonsense-mediated mRNA decay: degradation of defective transcripts is only part of the story. Annu. Rev. Genet. 49, 339–366. 10.1146/annurev-genet-112414-054639 26436458 PMC4837945

[B18] Henne W. M. Buchkovich N. J. Emr S. D. (2011). The ESCRT pathway. Dev. Cell. 21 (1), 77–91. 10.1016/j.devcel.2011.05.015 21763610

[B19] Hsu C. Morohashi Y. Yoshimura S. Manrique-Hoyos N. Jung S. Lauterbach M. A. (2010). Regulation of exosome secretion by Rab35 and its GTPase-activating proteins TBC1D10A-C. J. Cell. Biol. 189 (2), 223–232. 10.1083/jcb.200911018 20404108 PMC2856897

[B20] Kim K. M. Abdelmohsen K. Mustapic M. Kapogiannis D. Gorospe M. (2017). RNA in extracellular vesicles. Wiley Interdiscip. Rev. RNA 8 (4). 10.1002/wrna.1413 PMC5474163 28130830

[B21] Kowal J. Arras G. Colombo M. Jouve M. Morath J. P. Primdal-Bengtson B. (2016). Proteomic comparison defines novel markers to characterize heterogeneous populations of extracellular vesicle subtypes. Proc. Natl. Acad. Sci. U. S. A. 113 (8), E968–E977. 10.1073/pnas.1521230113 26858453 PMC4776515

[B22] Maquat L. E. (2004). Nonsense-mediated mRNA decay: splicing, translation and mRNP dynamics. Nat. Rev. Mol. Cell. Biol. 5 (2), 89–99. 10.1038/nrm1310 15040442

[B23] Mathieu M. Nevo N. Jouve M. Valenzuela J. I. Maurin M. Verweij F. J. (2021). Specificities of exosome versus small exosome secretion revealed by live intracellular tracking of CD63 and CD9. Nat. Commun. 12 (1), 4389. 10.1038/s41467-021-24384-2 34282141 PMC8289845

[B24] Melo S. A. Luecke L. B. Kahlert C. Fernandez A. F. Gammon S. T. Kaye J. (2015). Glypican-1 identifies cancer exosomes and detects early pancreatic cancer. Nature 523 (7559), 177–182. 10.1038/nature14581 26106858 PMC4825698

[B25] Meng Q. Chen C. Yang N. Gololobova O. Shi C. Dunn C. A. (2023). Surfaceome analysis of extracellular vesicles from senescent cells uncovers uptake repressor DPP4. Proc. Natl. Acad. Sci. U. S. A. 120 (43), e2219801120. 10.1073/pnas.2219801120 37862381 PMC10614838

[B26] Morelli A. E. Larregina A. T. Shufesky W. J. Sullivan M. L. Stolz D. B. Papworth G. D. (2004). Endocytosis, intracellular sorting, and processing of exosomes by dendritic cells. Blood 104 (10), 3257–3266. 10.1182/blood-2004-03-0824 15284116

[B27] Mulcahy L. A. Pink R. C. Carter D. R. (2014). Routes and mechanisms of extracellular vesicle uptake. J. Extracell. Vesicles 3. 10.3402/jev.v3.24641 PMC4122821 25143819

[B28] Nakase I. Futaki S. (2015). Combined treatment with a pH-sensitive fusogenic peptide and cationic lipids achieves enhanced cytosolic delivery of exosomes. Sci. Rep. 5, 10112. 10.1038/srep10112 26011176 PMC4443764

[B29] Notaras M. Allen M. Longo F. Volk N. Toth M. Li Jeon N. (2020). UPF2 leads to degradation of dendritically targeted mRNAs to regulate synaptic plasticity and cognitive function. Mol. Psychiatry 25 (12), 3360–3379. 10.1038/s41380-019-0547-5 31636381 PMC7566522

[B30] Ostrowski M. Carmo N. B. Krumeich S. Fanget I. Raposo G. Savina A. (2010). Rab27a and Rab27b control different steps of the exosome secretion pathway. Nat. Cell. Biol. 12 (1), 19–30. 10.1038/ncb2000 19966785

[B31] Perez-Hernandez D. Gutierrez-Vazquez C. Jorge I. Lopez-Martin S. Ursa A. Sanchez-Madrid F. (2013). The intracellular interactome of tetraspanin-enriched microdomains reveals their function as sorting machineries toward exosomes. J. Biol. Chem. 288 (17), 11649–11661. 10.1074/jbc.M112.445304 23463506 PMC3636856

[B32] Radulovic M. Stenmark H. (2018). ESCRTs in membrane sealing. Biochem. Soc. Trans. 46 (4), 773–778. 10.1042/BST20170435 29903934

[B33] Ratajczak J. Wysoczynski M. Hayek F. Janowska-Wieczorek A. Ratajczak M. Z. (2006). Membrane-derived microvesicles: important and underappreciated mediators of cell-to-cell communication. Leukemia 20 (9), 1487–1495. 10.1038/sj.leu.2404296 16791265

[B34] Rocha S. Carvalho J. Oliveira P. Voglstaetter M. Schvartz D. Thomsen A. R. (2019). 3D cellular architecture affects MicroRNA and protein cargo of extracellular vesicles. Adv. Sci. (Weinh) 6 (4), 1800948. 10.1002/advs.201800948 30828519 PMC6382357

[B35] Savina A. Vidal M. Colombo M. I. (2002). The exosome pathway in K562 cells is regulated by Rab11. J. Cell. Sci. 115 (Pt 12), 2505–2515. 10.1242/jcs.115.12.2505 12045221

[B36] Svensson K. J. Christianson H. C. Wittrup A. Bourseau-Guilmain E. Lindqvist E. Svensson L. M. (2013). Exosome uptake depends on ERK1/2-heat shock protein 27 signaling and lipid Raft-mediated endocytosis negatively regulated by caveolin-1. J. Biol. Chem. 288 (24), 17713–17724. 10.1074/jbc.M112.445403 23653359 PMC3682571

[B37] Thery C. Regnault A. Garin J. Wolfers J. Zitvogel L. Ricciardi-Castagnoli P. (1999). Molecular characterization of dendritic cell-derived exosomes. Selective accumulation of the heat shock protein hsc73. J. Cell. Biol. 147 (3), 599–610. 10.1083/jcb.147.3.599 10545503 PMC2151184

[B38] Van Niel G. Charrin S. Simoes S. Romao M. Rochin L. Saftig P. (2011). The tetraspanin CD63 regulates ESCRT-independent and -dependent endosomal sorting during melanogenesis. Dev. Cell. 21 (4), 708–721. 10.1016/j.devcel.2011.08.019 21962903 PMC3199340

[B39] Verweij F. J. van Eijndhoven M. A. Hopmans E. S. Vendrig T. Wurdinger T. Cahir-McFarland E. (2011). LMP1 association with CD63 in endosomes and secretion via exosomes limits constitutive NF-κB activation. EMBO J. 30 (11), 2115–2129. 10.1038/emboj.2011.123 21527913 PMC3117644

[B40] Xu R. Greening D. W. Zhu H. J. Takahashi N. Simpson R. J. (2016). Extracellular vesicle isolation and characterization: toward clinical application. J. Clin. Investig. 126 (4), 1152–1162. 10.1172/JCI81129 27035807 PMC4811150

[B41] Yim N. Ryu S. W. Choi K. Lee K. R. Lee S. Choi H. (2016). Exosome engineering for efficient intracellular delivery of soluble proteins using optically reversible protein-protein interaction module. Nat. Commun. 7, 12277. 10.1038/ncomms12277 27447450 PMC4961865

[B42] Zhou B. Xu K. Zheng X. Chen T. Wang J. Song Y. (2020). Application of exosomes as liquid biopsy in clinical diagnosis. Signal Transduct. Target Ther. 5 (1), 144. 10.1038/s41392-020-00258-9 32747657 PMC7400738

[B43] Zlotogorski-Hurvitz A. Dayan D. Chaushu G. Korvala J. Salo T. Sormunen R. (2015). Human saliva-derived exosomes: comparing methods of isolation. J. Histochem Cytochem 63 (3), 181–189. 10.1369/0022155414564219 25473095 PMC4340734

