# Supplementary figures and images for: Impact of UPF2 on the levels of CD81 on extracellular vesicles
Source: Front Cell Dev Biol. 2024 Nov 25;12:1469080. doi: 10.3389/fcell.2024.1469080 (PMC11625909; doi:10.3389/fcell.2024.1469080)

**A**

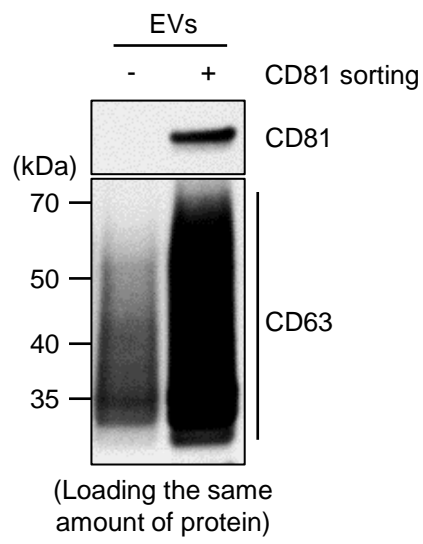

**B**

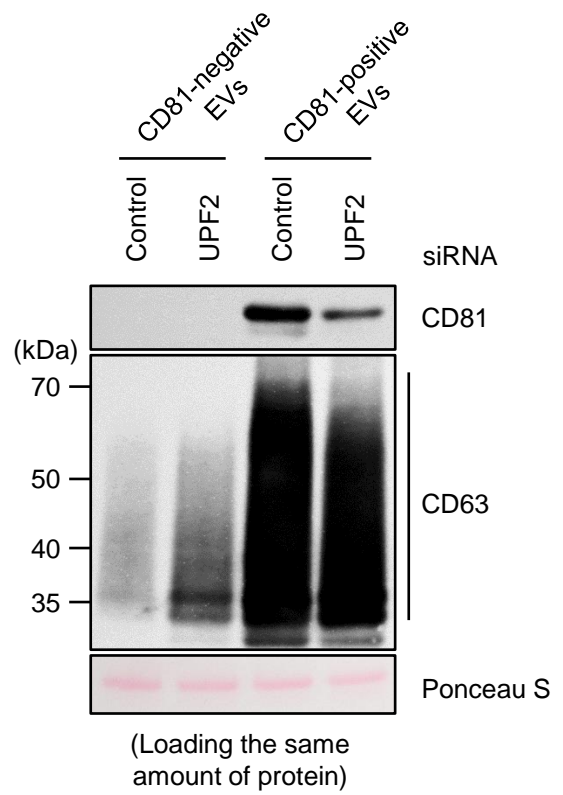

Supplement: Supplementary file 1 [file Image2.pdf]

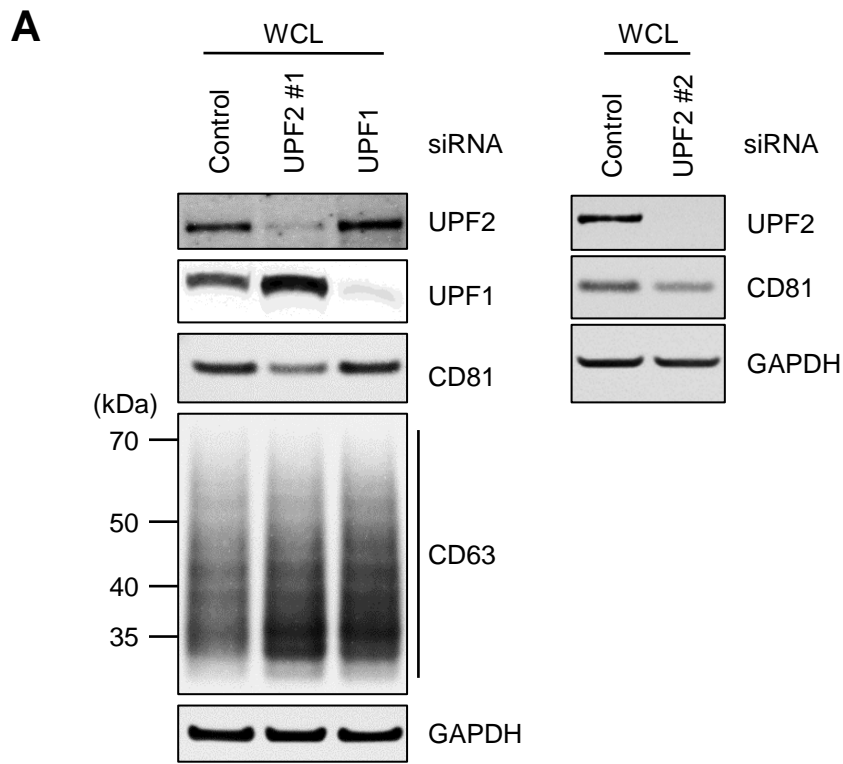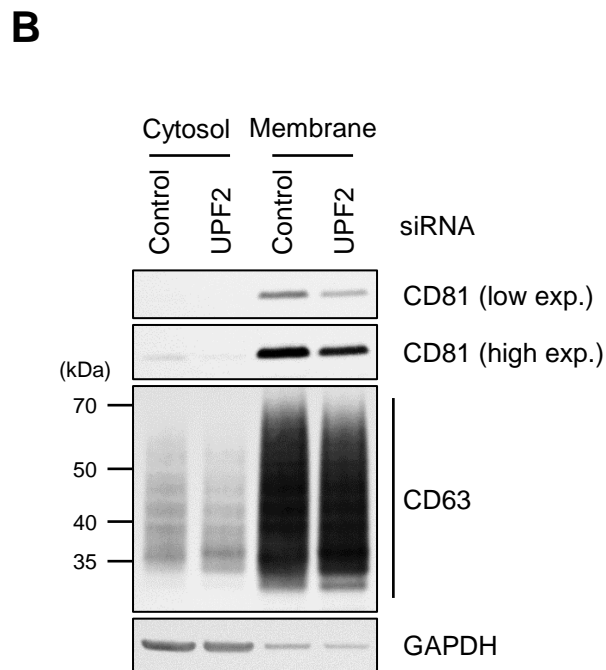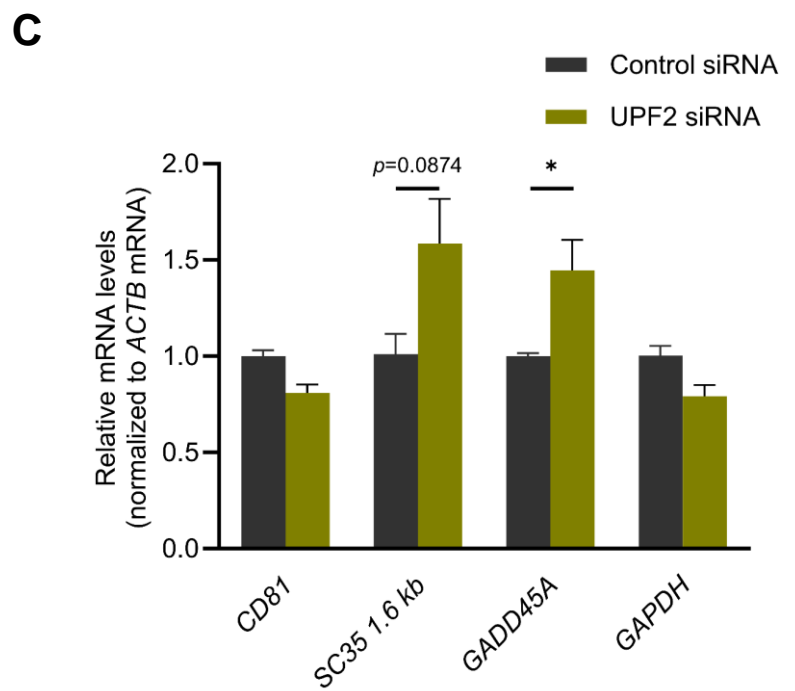

Supplement: Supplementary file 4 [file Image1.pdf]
