# Supplementary material for: Impact of UPF2 on the levels of CD81 on extracellular vesicles
Source: Front Cell Dev Biol. 2024 Nov 25;12:1469080. doi: 10.3389/fcell.2024.1469080 (PMC11625909; doi:10.3389/fcell.2024.1469080)
Supplement: Supplementary file 3 [file DataSheet1.docx]

**Impact of UPF2 on the levels of CD81 on extracellular vesicles**

Chaehwan Oh^1^, Krystyna Mazan-Mamczarz^2^, Myriam Gorospe^2^, Ji Heon Noh^3,*^, and Kyoung Mi Kim^1,*^

^1^ Department of Biological Sciences, Chungnam National University, Daejeon 34134, Republic of Korea

^2^ Laboratory of Genetics and Genomics, National Institute on Aging Intramural Research Program, National Institutes of Health, Baltimore, Maryland, USA

^3^ Molecular Aging Biology Laboratory (MABL), Department of Biochemistry, College of Natural Science, Chungnam National University, Daejeon 34134, Republic of Korea

^*^Correspondence:

Kyoung Mi Kim, Ji Heon Noh

Chungnam National University

99 Daehak-ro, Youseong-gu, Daejeon, 34134, Republic of Korea

Tel: +82-42-821-5498; Fax: +82-42-822-9690

Email: [km.kim@cnu.ac.kr](mailto:km.kim@cnu.ac.kr), journi@cnu.ac.kr

Running title: Impact of UPF2 on EVs

**SUPPLEMENTAL MATERIALS AND METHODS**

**Isolation of cytosolic and membrane proteins**

Cytosolic and membrane proteins were separated using a Mem-PER™ Plus Membrane Protein Extraction Kit, according to the manufacturer’s protocol (Thermo Fisher Scientific).

**Separation of CD81-positive and -negative EVs by magnetic-activated cell sorting (MACS)**

CD81-positive and -negative EVs were isolated from total EVs using the exosome isolation kit CD81 (Miltenyi Biotec, Germany) and MACS system according to the manufacturer’s instructions. Briefly, total EVs were magnetically labeled and loaded onto a μ Column, which was placed in the magnetic field of a μMACS™ Separator (Miltenyi Biotec, Germany).

**RNA isolation, reverse transcription, and quantitative real time PCR analysis**

Total RNA was extracted using the TRI™ Reagent solution (Invitrogen). After ethanol precipitation, the samples were treated with DNase I (Thermo Fisher Scientific). cDNA was synthesized using Random Hexamer Primer (Thermo Fisher Scientific) and RevertAid Reverse Transcriptase (Thermo Fisher Scientific). RT-qPCR analysis was performed using gene-specific primers, KAPA SYBR® FAST (Kapa Biosystems, UK), and QuantStudio Real-Time PCR instrument (Applied Biosystems, USA). The sequences of the specific primers used for qPCR are listed in **Supplementary TABLE**.

**SUPPLEMENTAL FIGURE LEGENDS**

**Figure S1. Effect of NMD factor deficiency on CD81 expression in HeLa cells.** (A) HeLa cells were transfected with control, UPF2 #1 (targeting the open reading frame region of UPF2, *left*), UPF2 #2 (targeting the 3’ untranslated region of UPF2, *right*) or UPF1 siRNAs, and EVs markers were assessed using Western blot analysis. (B) The expression levels of CD81 were assessed in cytosolic and membrane lysates using Western blot analysis. (C) Steady-state levels of *CD81* mRNA were quantified by RT-qPCR analysis and normalized to *ACTB* mRNA levels; UPF2 and UPF1, NMD factors; CD81 and CD63, EVs markers; GAPDH, a loading control or cytosolic marker; *SC35 1.6 kb* and *GADD45A* mRNA, NMD substrates; *GAPDH* mRNA, a negative control. Data in (C) represent the mean ± S.E.M. of three independent experiments. **P* ≤ 0.05.

**Figure S2.** **Knockdown of UPF2 decreases the secretion of CD81-positive EVs and increases the secretion of CD81-negative EVs.** (A) Total EVs were separated into CD81-positive and -negative EVs using MACS, and the expression levels of CD81 and CD63 were analyzed by Western blot analysis. (B) Total EVs derived from Control or UPF2 knockdown HeLa cells were separated as CD81-positive and -negative EVs using MACS. The separated EVs were loaded with same amounts of protein and the expression levels of CD81 and CD63 in separated EVs were determined by Western blot analysis.
